# Text message surveillance for rapid *salmonella* outbreak detection: a novel public health approach

**DOI:** 10.1017/S0950268825100617

**Published:** 2025-09-29

**Authors:** Neil Franklin, Kirsty Hope, Kathryn Glass, Martyn Kirk

**Affiliations:** 1 National Centre for Epidemiology and Population Health, Research School of Population Health, The Australian National University, Canberra, ACT, Australia; 2 Health Protection NSW, NSW Ministry of Health, Sydney, NSW, Australia

**Keywords:** foodborne outbreaks, interview, public health follow-up, *salmonella*, SMS, surveillance

## Abstract

In large public health jurisdictions, only a small proportion of people infected with *Salmonella* are interviewed due to resource constraints. As such, sources of illness are rarely found, and preventative action not implemented. We trialled alternative methods to contact notified salmonellosis cases to collect information on exposures and risks, focusing particularly on the feasibility of SMS (short message service)-based surveillance. Over five-years period we sequentially mailed letters, sent online surveys, and then text messages. The SMS approach was designed to assess the efficiency of a two-way personalized messaging model in gathering actionable public health data. The personalized SMS-follow-up model demonstrated the highest success: 56% of cases responded, enabling the identification and intervention of 10 distinct point-source outbreaks of *Salmonella.* SMS-based surveillance offers a novel, efficient, and acceptable method for collecting critical food exposure data in *Salmonella* cases. In settings where resources are constrained, SMS can complement traditional case follow-up methods, enhancing both the timeliness and effectiveness of outbreak detection. Integrating this follow-up with routine clinical care could further enhance the acceptance and success of this method. This study highlights the promise of SMS in streamlining surveillance efforts and warrants further exploration for application to other infectious diseases.

## Introduction

*Salmonella* is one of the most common causes of foodborne illness. In New South Wales (NSW), typically over 3000 infections are reported to the health department annually [1]. Interviewing individuals infected with *Salmonella* may identify common sources of infection and lead to preventive actions. An interview of a salmonellosis case involves a 20-page questionnaire that explores the nature of their illness and all possible exposures, which may take up to an hour to complete. In NSW, general practitioners (GP) or hospital doctors diagnose *Salmonella*, and cases are usually only interviewed by a public health officer if they are found to be part of a cluster or outbreak. We estimate that only 5–15% of salmonellosis cases are followed up in NSW [2].

When public health unit staff interview cases, there is usually a delay between a person’s illness and the interview. If a case is found to be part of a cluster by whole genome sequencing (WGS), the interview is often conducted up to 4 weeks after the illness. This results in poorer recall of the foods consumed in the period of most concern. In a study from 2016, epidemiologists simulated food recall in an outbreak investigation, comparing recall with cashless cafeteria sales records [3]. They found false-negative recall was twice as high 3 weeks after than it had been at 1 week.

Due to limited follow-up and poor timeliness of interviews, the source for most salmonellosis cases is never identified, and necessary preventative public health interventions are not implemented. To address these issues, NSW Health protection trialled new methods to contact salmonellosis cases and gather risk information without adding to the workload of public health staff. The project began at the end of 2013, initially using mailed letters and invitations to online surveys. By 2015, 74% of Australian adults owned smartphones [4], making SMS an increasingly viable data collection method. The aim was to test the acceptability and usefulness of each method and to recommend a plan for scaled up salmonellosis investigation.

SMS-based communication has shown promise in delivering healthcare interventions [5] and in emerging disease outbreaks [6]. However, reports of its utility in routine public health surveillance, particularly for notifiable disease follow-up, remains rare in the published literature. Many public health agencies face similar challenges in timely surveillance and outbreak detection from foodborne pathogens. This study provides insights into how mobile technologies can be leveraged for public health practice to enhance disease surveillance and improve outbreak detection efficiency across diverse healthcare systems.

## Methods

### Salmonella surveillance in NSW

In NSW, doctors and laboratories are required to notify cases of human salmonellosis to the NSW Public Health system. Local Public Health Unit (PHU) staff manage individual notifications of cases and local outbreaks, while the NSW Health department staff manage state-wide surveillance and outbreak coordination. Typically, only salmonellosis cases associated with outbreaks and cases infected with high-priority *Salmonella* serovars (based on public health significance and other internal surveillance priorities) are required to be followed up. In NSW, WGS is routinely done for the *Salmonella* serovars *typhimurium* and *enteritidis*, which make up between 60% and 40% of all cases. This project was managed by NSW Health with local support provided by the PHUs.

### Approach to case follow-up

This project had three phases of development occurring during each seasonal summer peaks (~4 months long) of *Salmonella* notifications in 2013 to 2015, prior to the main phase that ran continuously from 2017 to 2020 (29 months).

Salmonellosis cases notified to NSW Health by electronic reporting from laboratories were included in all phases of the project. We excluded cases under the age of 2 years old, notifications relating to detection in non-faecal specimens, notifications that indicated overseas acquisition as reported by the doctor on the laboratory request, and notifications already flagged for interviewing. Prior to project commencement, information was sent to NSW public health units and to general practitioners to make them aware of the new process. Electronic Laboratory Report (ELR) notifications were used because these are reported to NSW Health directly from the diagnostic laboratories faster than other notification methods, and an aim of the project was to contact *Salmonella* cases as soon as possible after their illness onset.

**Figure 1.**
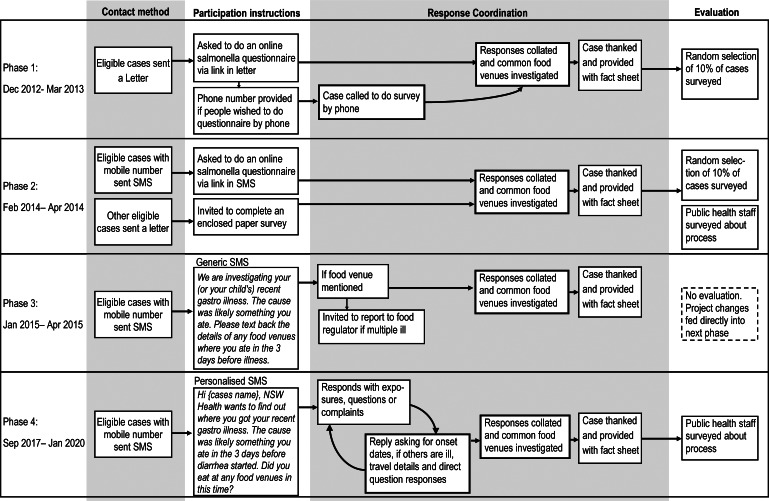
Methodology flow chart for the four phases of the project.

For all phases of the project, eligible cases were either sent a letter or SMS inviting them to complete a *Salmonella* questionnaire online or to respond directly with the SMS. Figure 1 details the different methods of contacting cases, the questions asked in each phase, and response procedures involved over the course of the four phases. The main progression of the project was from a letter inviting cases to complete an online survey, then to complete the same survey via a SMS, then asking cases to respond to a single question via SMS, ending with a personalized and more realistic question and answer SMS conversation. Across all phases, cases received a *Salmonella* fact sheet (hard copy or via link), to ensure they received appropriate advice on prevention.

### Evaluation

An evaluation was conducted after each of the first two phases to establish the acceptability of contacting salmonellosis cases via these methods. We randomly selected approximately 10% of eligible participants to participate in the evaluation. Selected cases were given a questionnaire over the phone by a single interviewer. Public health staff who would have normally been responsible for *Salmonella* follow-up were also interviewed to find out about their experience and perspective on the project in phases 2 and 4. There was no evaluation of phase 3 conducted, as improvements in the project were fed directly into phase 4.

### Short message service (SMS)

SMS messages were sent, as described in Figure 1, through a web-based electronic messaging service (https://www.prodocom.com.au), and responses received from cases were recorded in an individual disease record in the notifiable disease record system and within an excel spreadsheet.

If the information provided by a case was immediately identified as being linked to another case (e.g. two SMS responses concurrently mentioned the same food establishment), or the response indicated a cluster of illness among a group of diners who ate the same meal, details of the cases were forwarded to the PHU to undertake further investigation. The NSW Food Authority was also informed when a cluster of illness appeared to relate to a specific venue. The information obtained through SMS responses was also combined with other *Salmonella* case investigation methods (such as traditional case interviews, complaints made to the NSW Food Authority, and geographical clustering), to explore common exposure risks. If a case requested a call back, the details were forwarded to the relevant PHU.

### Analysis

Data were entered into STATA. Chi-squared tests were used to test differences in response rate by demographic factors. This project involved collecting risk factor information for public health investigation under the NSW Public Health Act [7], so review by a human research ethics committee was not required.

## Results

Table 1 summarizes the results of the four phases of the project. Response rates for interviews increased with each phase from 22.2% in 2013 to 55.9% in 2020. The use of SMS in the final two phases meant cases were contacted and asked about exposures a median 5 days after their specimen was taken. Public health salmonellosis control action involving an investigation with the state food regulators was initiated based on responses in all but the first phase.

**Table 1.** Summary of *Salmonella* contacts and responses for each phase of the project

Project phase	*Salmonella* cases in NSW, *n*	Eligible cases invited, *n* (% of total)	Responses from those contacted, *n* (%)	Days from specimen date to contact, *n* (median (range))	Responses resulting in intervention, *n* (%)
Phase 1: December 2012–March 2013	1440	Letter: 275 (19.1%)	61 (22.2%) Online: 48 Phone: 13	10^a^ (7–32)	0 (0%)
Phase 2: February 2014–April 2014	1487	Online: 638 (42.9%) Letter: 63 (4.2%)	199 (28.4%) Online: 186 (29.2%) Letter: 13 (20.6%)	Online: 8 (4–34) Letter: 17^a^ (11–33)	18 (9.0%)
Phase 3: January 2015–April 2015	1552	SMS: 475 (30.6%)	218 (45.9%)	5 (1–33)	15 (6.9%)
Phase 4: September 2017–January 2020	7931	SMS: 2197 (27.7%)	1201 (55.9%)	5 (1–30)	33 (2.7%)

a Four days added to date letter sent to account for average letter transport times as reported by Australia post (3–5 days).

## Phases 1 and 2 evaluation

In phase 1, we interviewed 28 (10.2%) cases. Three people reported not receiving the mailed invitation. Only cases who had received the letter prior to receiving their salmonellosis diagnosis from their GP (7%) and those who reported being very unwell (7%) expressed concern about the letter. Those who completed the survey reported few issues. The main reasons for non-completion of the survey included lack of time (24%), not considering it important (24%), asked to be called instead (24%), access issues (17%), or no recall of the foods eaten (12%). Respondents felt they would be more likely to complete the survey if offered by telephone call (89%), on paper by return mail (64%), or by email (50%) than the letter inviting them to go online.

In phase 2, we interviewed 78 (11.1%) cases. Thirty-one per cent of cases learned about their *Salmonella* diagnosis through the SMS prior to their GP informing them; 40% of these cases expressed concern about this as a means of notification, which was higher than the rate of concern for those who had been informed by their GP (11%). Despite high smartphone ownership (87%), only 12% completed the survey on their phone, with most (65%) copying the link provided into their desktop or laptop computer. Those who completed the survey reported few issues, though many (46%) expressed a desire for feedback after completing the survey. The main reasons for non-completion of the survey included lack of time (38%), access issues (21%), forgetfulness (14%), or received a call from the PHU instead (10%). In contrast with the phase 1 evaluation, respondents reported a preference for doing the survey via the internet (53%), then by email (32%) followed by telephone call (17%). However, when asked if they would respond to a direct SMS asking for less information instead, most people (95%) said that they would respond.

## Phase 4 results

During the phase 4 analysis period of 1 September 2017–10 January 2020, there were 7945 notifications of *Salmonella* in residents of NSW. Of the notified cases, 2197 (28%) were found to be eligible to be sent an SMS. The predominant reasons for cases not progressing to the SMS project was not having a mobile phone number on file (37.9%), the PHU already following up the case (24.8%), or the case being too young for inclusion in the project (19.1%).

Of the 2197 eligible for SMS, the messaging software reported 2150 (98.0%) of the SMS were successfully sent. Of these, 1201 (55.9%) cases responded to the SMS. Response rates were generally consistent across demographic factors, except that people who were 40 years and older were more likely to respond than people under 40 (58.6% vs. 53.8%, *p* = 0.027). Females had higher response rates than males (57.8% vs. 53.6%, *p* = 0.051), but this was not statistically significant.

Most cases responded to the SMS with a possible exposure (78.4%), the largest portion of people reported food venues that they ate at from outside the home (38.3%) followed by people who reported travel in the incubation period (26.1% overseas travel and 0.2% interstate travel). A minority of cases (12.4%) requested to be called instead and a small proportion (3.7%) either did not have a gastrointestinal illness or had ongoing gastrointestinal issues so could not report on the period in question.

Most people (92.9%) replied to the message within 24 h of receiving it. The median time between specimen date and replying to the SMS was 5 days. Some people (31.8%) were sent a follow-up question asking for additional information, of these, 88.7% replied. There were 62 people (5.9%) who reported that they knew of other people who were also ill from consuming the same food.

There were 33 respondents (7.2% of respondents who reported food venues) who were ultimately linked to 16 *Salmonella* outbreaks during the analysis period. Ten of these outbreaks were discovered solely due to the SMS responses. The other cases were found to be linked to *Salmonella* outbreaks already under investigation. All of these outbreaks led to food safety investigations at the identified food premises.

There were 19 (1.6%) cases who responded questioning the validity of the SMS they had received. SMS responses from five (0.4%) cases expressed displeasure at having received an SMS about their illness in place of a phone call.

## Phase 4 evaluation

At the end of the project, some PHU staff interviewed noted that the project had increased the workload, as a significant number of cases had requested a call instead of communicating via SMS. Additionally, some of these did so just to establish the validity of the message. Typically, PHUs that did not do regular *Salmonella* follow-up prior to the project found the project increased their workload, whereas PHUs that already did some *Salmonella* follow-up found the project decreased their workload by providing certain info by SMS that they would have otherwise had to call to retrieve, such as overseas travel information.

## Discussion

Reducing the burden of salmonellosis is challenging due to high case numbers and limited public health resources for follow-up interviews. This project demonstrated that public health agencies can gather risk information promptly and efficiently by using personalized SMS to communicate with *Salmonella* cases. Importantly, this approach adds minimal burden to the workload of public health unit staff, particularly those without capacity to conduct full *Salmonella* interviews. The high response rate (55.9%) in the final stage suggests strong acceptability among cases, particularly when personalized messaging techniques were used.

The success of SMS follow-up lies in its ability to minimize recall bias by reducing the time between illness and interview. Traditional case interviews are often conducted weeks after infection, leading to incomplete food exposure histories. In the absence of routine interviews, molecular typing is required to identify *Salmonella* clusters and initiate investigations, but this typing can be received many weeks after the illness. Prior research has shown that recall error increases as time to interview lengthens, with false-negative rates nearly doubling after 3 weeks [3]. In this study, SMS responses were obtained within a median of 5 days after specimen collection, enhancing the reliability of reported exposures. Importantly, 10 outbreaks were identified solely due to SMS responses, enabling public health interventions before genetic clustering had flagged these events. In one outbreak detected through this project, SMS responses from three separate cases linked their illness to a chain of bakery stores with 10 outlets. As a result, the food safety authorities intervened 3 weeks before molecular typing would have detected the cluster. This undoubtedly resulted in far fewer cases than if the outbreak had been detected by conventional means [1].

It is important to note this project predates the COVID-19 pandemic – a period during which digital tools for public health underwent significant changes. In Australia, COVID-19 laboratory results were received by SMS and much of the follow-up of COVID-19 cases and contacts was also done via bulk SMS services [8]. Consequently, the public are now more accustomed to receiving text messages from government and private health services. Considering this, people may be even more willing to engage in a project like this because they now see it as part of normal clinical follow-up.

The digital health space has used SMS with high patient satisfaction [9] to successfully provide appointment reminders [10], enhance clinical adherence [11], and improve clinical outcomes [12]. SMS is most often used to follow-up patients already enrolled in a treatment programme or study or for the distribution of educational material. The use of SMS to gather routine public health risk information remains rare in the published literature. A recent active surveillance study using SMS to measure childhood diarrhoea in volunteers found a similar response rate to our study at 47%, they also found that fewer questions and incentivization increased response rates to approximately 51% [13]. Our study similarly found subsequent replies also dropped off and so recommend keeping the conversation short and with the aim of collecting the most important pieces of information first. We began by asking about food venues, then overseas travel. Future expansions of the project could explore continued messaging to ask about the next most important risk factor, which could be adapted to current issues (e.g., if an active outbreak is investigating a specific exposure). However, we would expect that responses rates will drop with each additional message, so strategies to maintain user engagement would also need to be considered.

There were potential limitations to the SMS follow-up approach used in this study. First, non-response bias may have affected the representativeness of the data, as individuals without mobile phones or those distrustful of SMS were excluded. Previous studies on SMS-based interventions in clinical care have reported similar biases, with marginalized groups being less likely to engage [14]. Second, the reliance on self-reported food exposures means recall bias may be present, despite the shorter time to interview.

Another key limitation is the existence of SMS scams, which may lead to distrust of unsolicited messages. Some cases questioned the validity of the SMS, highlighting the need for strategies to increase public confidence. This study addressed these concerns by incorporating recipient names, official public health contact details, and offering call-back options. These elements distinguish the third and fourth phases of the project, with the increase in response rate from 46% to 55% potentially reflecting the effectiveness of these techniques. Future implementations should consider integrating official branding, authentication links, or additional verification methods such as integration with government health portals to further enhance credibility. Policymakers should establish clear regulatory frameworks to govern the ethical use of SMS in public health, addressing privacy concerns, and reinforcing transparency in data collection. Finally, while this study demonstrated success in Australia, its applicability in other countries with different health systems and regulations should be explored.

Although some cases expressed concerns about receiving an SMS about a notifiable disease, few cases reported discomfort in project evaluations. This suggests that asking about food consumption for an enteric disease is not seen as highly sensitive. Studies using SMS for sexual health screening also report high acceptability [15]. The success of this approach in salmonellosis follow-up indicates that SMS-based surveillance could be extended to other diseases requiring rapid case follow-up. Future work should explore how best to integrate SMS into broader public health strategies while ensuring efficiency and public trust.

We recommend that public health agencies build on this project and incorporate newer messaging technology to enhance collection of risk factor information for salmonellosis. This could include interactive two-way messaging, links to surveys which could broaden the breadth of risk information collected, and targeted health communications. Interactive two-way messaging, as used in this project, was an effective way to elicit a response. Incorporating a link to a survey which could be optionally completed would increase the information that can be collected and allow consistent data capture. Even if a case does not want to reply to a message with information, there is still value in providing a link to public health information about their illness. This is especially true for a disease like salmonellosis, which can have serious consequences but is too common for public health staff to give individual attention to every case. Engaging GPs in the process would also be highly beneficial for the follow-up. Not only would the combined care information benefit the *Salmonella* case, but the GP could also prime the case to expect the text message and start thinking about risk factors to report.

## Conclusion

Given the increasing number of preventable communicable disease notifications and increasingly stretched public health resources, new methods for gathering risk information are required. Two-way text messaging is an acceptable and efficient means to collect food exposure information for *Salmonella* cases that is also able to detect outbreaks. Future work should focus on optimizing SMS strategies, expanding their application to other diseases, and integrating them with the clinical care giver into routine public health practice.

## Data Availability

The data used in this study were obtained under the provisions of the NSW Public Health Act 2010. Due to privacy, confidentiality, and legislative restrictions, these data cannot be shared publicly.
